# Checklist of the ants (Hymenoptera, Formicidae) of the Solomon Islands and a new survey of Makira Island

**DOI:** 10.3897/zookeys.257.4156

**Published:** 2013-01-14

**Authors:** Eli M. Sarnat, Benjamin Blanchard, Benoit Guénard, John Fasi, Evan P. Economo

**Affiliations:** 1 Antwork Consulting, LLC, PO Box 563 Happy Camp, CA 96039 USA; 2 Department of Entomology, University of Illinois, Urbana, Illinois 61801; 3 Department of Ecology & Evolutionary Biology, University of Michigan, 830 N. University St., Ann Arbor, MI 48109 USA; 4 Okinawa Institute of Science and Technology, 1919-1 Tancha, Onna-son, Kunigami gun, Okinawa, Japan 904-0495; 5 Science Department, School of Education, Solomon Islands College of Higher Education, P O Box R113. Honiara. Solomon Islands

**Keywords:** Biogeography, checklist, Makira Island, Pacific Islands, Solomon Islands, species distributions, taxonomy, Formicidae

## Abstract

The intent of this paper is to facilitate future research of the Solomon Islands ant fauna by providing the first comprehensively researched species inventory in over 75 years. The species list presented here includes the names of all ant species recorded from the islands that are available in the literature together with specimen records from several museum collections and new records from our 2008 Makira field expedition. All the names of described species presented are valid in accordance with the most recent Formicidae classification. In total, the checklist is composed of 237 species and subspecies (including 30 morphospecies) in 59 genera representing nine subfamilies. We report that the recent field expedition added 67 new species records to Makira and 28 new species records to the Solomon Islands. Our research recovered species occurrence records for 32 individual islands and five island groups. The five islands with the highest number of recorded species are: Makira (142 spp.), Guadalcanal (107 spp.), Malaita (70 spp.), Santa Isabel (68 spp.), and Rennell (66 spp.). Based on our results, we discuss the taxonomic composition of the archipelago’s ant fauna, which islands are most in need of additional sampling, and the importance of establishing biodiversity baselines before environmental threats such as the invasive ant *Wasmannia auropunctata* cause irrevocable harm to the native biodiversity.

## Introduction

The intent of this paper is to facilitate future research of the Solomon Island ant fauna and that of the larger Pacific Island region by providing the first comprehensively researched species list in over 75 years (Mann 1919; Wheeler 1935b). Reliable species lists are the foundation for biodiversity and biogeography research. This is especially true for archipelago systems such as the Solomons which serve as natural laboratories for studying the interface of geography, evolution and ecology (Diamond 1975; Diamond and Mayr 1976; Greenslade 1968; MacArthur and Wilson 1967; Mayr and Diamond 2001; Wilson 1959a; 1961). Accurate faunal lists at the archipelago level allow us to analyze biogeographic patterns at the regional scale, and faunal lists at the individual island level allow us to analyze more local scale patterns. These studies are crucial for the development of precise conservation plans that incorporate the distribution of endemic and rare taxa.

Faunal lists are also necessary for recognizing biodiversity blind spots and identifying which regions and islands are most in need of additional sampling. Increasing environmental threats such as deforestation, mining, agriculture and the spread of invasive species give urgency to surveying these poorly sampled regions. In order to assess how these threats affect native biodiversity, it is important to establish baseline inventories before local populations and endemic species are driven extinct.

## Geography, geology and climate

**Figure 1.**
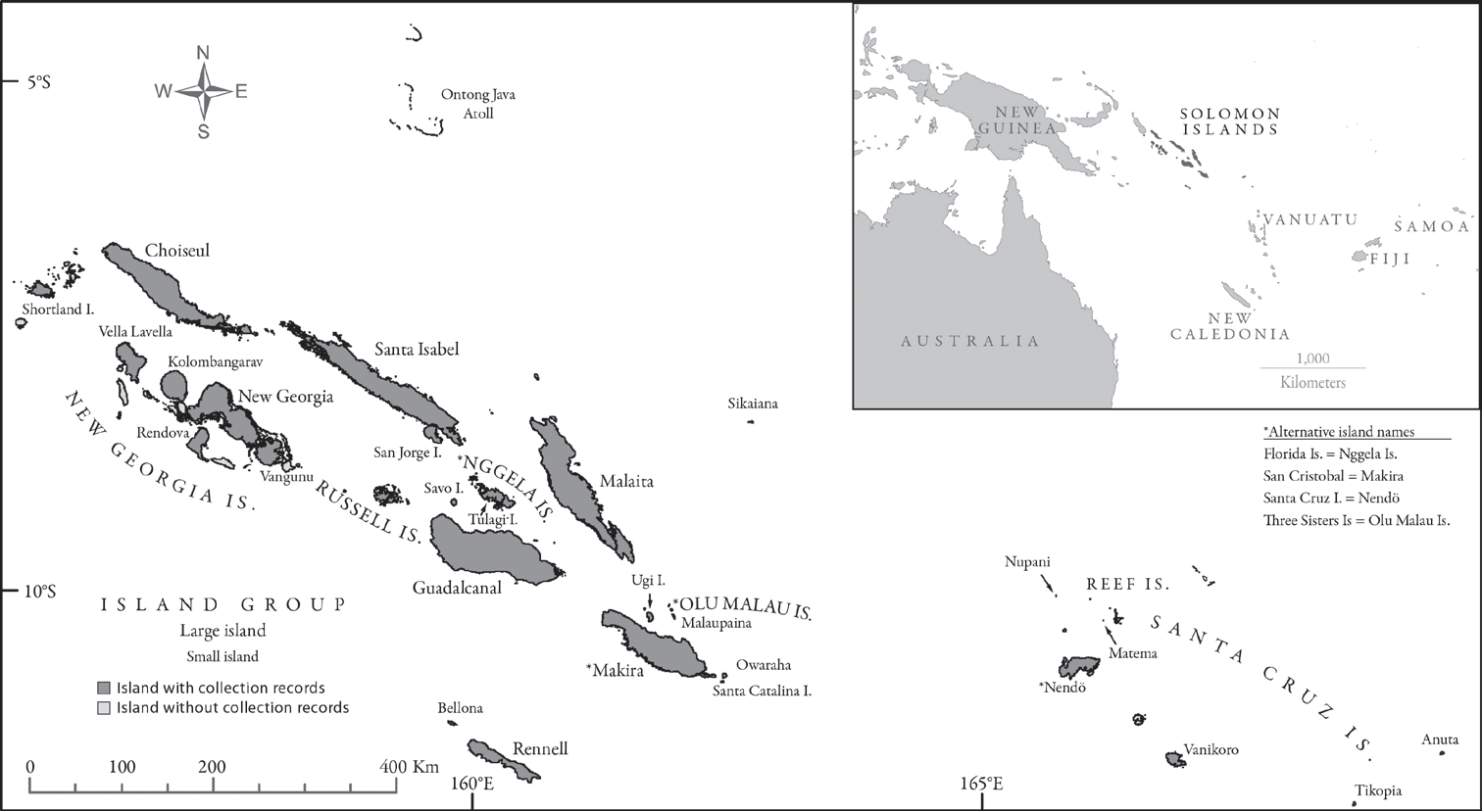
Map of the Solomon Islands. The map presents all islands and island groups for which ant species were recorded. Each island/island group from which ant species are known is labeled with the geographic name and filled darker grey. Islands for which no ant records appear in the literature are unlabeled and filled with lighter grey. Relevant historic island names from the colonial era are presented with their contemporary counterparts.

The Solomon Islands is a nation in the Southwest Pacific that is composed of seven large islands, a dozen mid-sized islands and over a thousand smaller islands (Figure 1). These islands, which comprise a total land area of 27,556 km^2^, are situated between the latitudes 5° and 13°S, and longitudes 155° and 169°E. The major central islands include the Shortlands, Choiseul, the New Georgias, Santa Isabel, the Russells, Guadalcanal, the Nggelas (Floridas), Malaita, Makira (San Cristóbal), and Olu Malau (Three Sisters). Rennell and Bellona are southern outlying islands situated along the northern margin of the Coral Sea Basin. Northern outlying islands include Sikaiana and the Ontong Java Atoll, which are on the southwestern edge of the Ontong Java Plateau. The eastern outlying islands of the Santa Cruz group are politically part of the Solomon Islands, but are geologically linked to the islands of Vanuatu (Kroenke and Rodda 1984).

The Solomons consist of a double chain of islands separating the Pacific Plate to the north from the Australian Plate to the south (Hall 2002). The islands are believed to have been formed entirely of oceanic origin, and there is no evidence that they were ever attached to continental systems or incorporated any terrains of continental origin (Kroenke and Rodda 1984). They are, in this sense, Darwinian Islands (Gillespie and Roderick 2002). According to several geologic models (Hall 2002) the Solomon Arc formed approximately 40 Ma as part of the Melanesian Arc system. It is unclear, however, when the islands emerged above sea level.

Bougainville, which lies to the west, belongs politically to Papua New Guinea but is geographically part of the Solomon Islands. The next closest neighbor nation is Vanuatu, which lies southeast of the main archipelago and nearly due south of the Santa Cruz Is.

The climate of the Solomon Islands is characterized as humid with a mean temperature of 27 °C (80 °F) and relatively few fluctuations of temperature or weather. The cooler and drier part of the year occurs from June through August, and the warmer and wetter season occurs from September through May. The annual rainfall is approximately 3050 mm (120 in).

## History of ant collection and research in the Solomon Islands

The first ants described from the Solomon Islands were authored by Forel (1910) in a paper on Australian ants based on the collections of W.M. Froggatt and Rowland Turner. Froggatt visited the Solomon Islands to study the insects of the coconut palms, and collected at Tulagi I. and in the Russell Group. W.M. Mann (1919) provided the first and only comprehensive revision of the Solomon Island ant fauna. He spent six months on the archipelago from 19 May to 24 November 1916, and collected on the islands Guadalcanal, Makira, Malaita, Malaupaina, New Georgia, Nggela Sule, Owaraha, Rendova, Russell Is., Santa Cruz, Santa Isabel, Tulagi and Ugi. Mann reported the occurrence of 136 currently recognized species and subspecies, of which he described 68 from his own collections. In addition to a broad discussion of the archipelago’s ant fauna, the treatise also includes keys to Melanesian species of *Anochetus*, *Crematogaster*, *Cryptopone*, *Eurhopalothrix*, *Leptogenys*, *Myrmecina*, *Triglyphothrix* (= *Tetramorium*), *Turneria*, and *Wheeleripone* (= *Gnamptogenys*).Additional relevant publications from Mann include descriptions of ant guests from Fiji and the Solomon Islands (Mann 1920), and accounts from his travels in the Solomon Islands (and elsewhere) in his book *Ant Hill Odyssey* (Mann 1948).

H. Viehmeyer (1924) described a new subspecies *Euponera (Mesoponera) melanaria* subsp.* manni* (= *Pachycondyla manni*) from Mann’s collections at the Museum of Comparative Zoology (MCZ). H. Donisthorpe (1941) described *Nylanderia manni* from a worker that was on the same pin as several workers of *Camponotus loa* Mann, all of which were labeled as *Iridomyrmex myrmecodiae* Emery (= *Philidris myrmecodiae*). Donisthorpe attributed the close similarity of all three species to mimicry.

W.M. Wheeler’s first contribution to the Solomon Island ant fauna was his description of *Opisthopsis manni* based on specimens collected by Mann from Malaupaina (Wheeler 1918). Wheeler (1934) later published on ants collected by Maurice Willows Jr. from the Santa Cruz and Danger Islands. He listed the names and collection records of 27 currently recognized taxa, including original descriptions for two species (*Nylanderia dichora*, *Stereomyrmex dispar*) and one subspecies (*Polyrhachis labella brunneipes*), along with the first published record of *Tapinoma melanocephalum* from the Solomons (Wetterer 2009). These records are combined with those of Forel and Mann in Wheeler (1935b).

William Brown treated many Solomon Island taxa in his revisions (Brown 1948; 1958a; b; 1960; 1975; 1976; 1978; 1988; 1995; Willey and Brown 1983). Gressitt (1958) reported on the pest behavior of *Iridomyrmex myrmecodiae* (= *Philidris myrmecodiae*) invading buildings in Malaita. According to Wilson (1962), the B.P. Bishop Museum, Honolulu, initiated a collecting program in the Solomons under the direction of Gressitt, and there is likely a considerable amount of ant material that remains unreported in the literature.

Research on economically important ants involved in coconut production was an active field in the Solomons from the 1930’s through the 1960’s (Leston 1973; Lever 1933; 1961; O’Conner 1949; 1950; Phillips 1940; 1956). E.S. Brown (1959) recorded over 60 species of ants (including five new country records) collected during his work among coconut plantations in Guadalcanal and Malaita.

Philip J.M. Greenslade has arguably collected more thoroughly across the Solomons than anyone since Mann. Greenslade published seven papers between 1964 and 1988 based on fieldwork he conducted in the Solomons (Greenslade 1964; 1971a; b; 1972; Greenslade and Greenslade 1970; 1971; 1977). The research focused primarily on the ecology of ants that are dominant in coconut plantations and are involved in the biological control of a coconut pest, *Amblypelta cocophaga* China and the premature nutfall of coconut fruit. In addition to providing valuable ecological information on the four most dominant ant species in these plantations (*Anoplolepis gracilipes* (Smith, F.), *Oecophylla smaragdina* Forel, *Pheidole megacephala* (Fabricius) and *Philidris cordata* (Smith, F.)), Greenslade also collected a broad diversity of less economically important ant species, mainly from Mt. Austen (Guadalcanal) and Kukum—the nearby Solomon Is. Department of Agriculture farm. These specimens, most of which were deposited at the ANIC, included many new species in addition to the first records of *Problomyrmex* (Taylor 1965) and *Colobostruma* (Bolton 2000) for the Solomon Islands. Interestingly, Greenslade’s (1968) work on the avifauna of the Solomon Islands was the first to apply the taxon cycle model to birds.

E.O. Wilson included many species from the Solomon Islands during his revisionary work of the Melanesian ant fauna, including species currently in the genera *Amblyopone*, *Leptogenys*, *Platythyrea* and *Stigmatomma* (1958a); *Ponera*, *Cryptopone*, *Hypoponera*, *Pachycondyla* and *Rhytidoponera* (1958b); *Anochetus* and *Odontomachus* (1959c); and *Cerapachys* (1959d). Wilson and Taylor (1967) added several new species records for the Solomons, including *Ponera incerta* (Wheeler) and *Strumigenys karawajewi* Brown (as *Strumigenys dubia* (Brown)). Wilson and Hunt (1967) included records for the Solomons. In addition to these taxonomic studies, Wilson also included ants from the Solomons in his influential papers on the taxon cycle hypothesis (Wilson 1959a; 1961) and the theory of island biogeography (MacArthur and Wilson 1967).

Wilson’s (1962) paper on the ants of Rennell and Bellona Islands examined specimens collected from three sources: a Danish Expedition (Wolff 1955); a British expedition (Bradley 1955), and a private collection made on Rennell and Bellona for several weeks during 1955, by Mr. E.S. Brown. Wilson recorded 25 species of ants in 17 genera from Rennell (including the first record of *Dilobocondyla* from the Solomons). He considered these to represent a large percentage of the actual ant diversity, but admitted that the lack of cryptobiotic ponerine and myrmicine species suggest that his list is incomplete. He concluded that the Rennell ant fauna is primarily composed of widespread Pacific natives that invaded the island relatively recently and are representative of ‘Stage-I’ species discussed in his taxon cycle hypothesis (Wilson 1959a; 1961).

Robert Taylor, in addition to describing *Problomyrmex salomonis* (Taylor 1965), also described *Eurhopalothrix greensladei* (Taylor 1968), and *Stigmatomma gnoma* ( = *Amblyopone gnoma*) (Taylor 1979) from specimens collected by P.J.M. Greenslade on or near Mt. Austen. Rudolf Kohout’s work on *Polyrhachis* added several new species records to the Solomons, introduced new synonyms and nomenclatural changes, and included the description of three new species (*Polyrhachis greensladei*, *Polyrhachis setosa*, *Polyrhachis undulata*) endemic to the Solomons (Kohout 1990; 1998; 2006). Barry Bolton described *Polyrhachis nofra* (Bolton 1975), from the Solomons, provided the replacement name of *Tetramorium mutatum* Bolton for the junior secondary homonym *Triglyphothrix* (= *Tetramorium*) *pulchella* Mann (Bolton 1985), and added new records of dacetines in the Solomons (Bolton 2000). Bolton(1976) also described *Tetramorium vombis* from specimens Mann (1919) mistakenly identified as *Tetramorium obesa* André. Kugler described *Rogeria megastigmatica* from a Greenslade collection made on Guadalcanal (Kugler 1994). Lattke included the Solomon Islands in his biogeographic analysis of *Gnamptogenys* in Southeast Asia (Lattke 2003) and described two new species (*Gnamptogenys preciosa* and *Gnamptogenys solomonensis*)from there (Lattke 2004). Lucky & Sarnat (2008) included *Lordomyrma epinotalis* Mann in their phylogenetic and biogeographic analysis of the genus. Sarnat and Moreau (2011) included *Pheidole* species from the Solomons in their phylogenetic and biogeographic analysis of the Fijian *Pheidole* and selected congeners from across the Pacific.

## Methods

### Compilation of names

In order to compile a comprehensive and accurate inventory of ant species recorded from the Solomon Islands, we researched taxonomic names that were associated with the region in the literature. We reviewed the names of all taxa that were originally described from Solomons, reviewed specimen records from Antweb.org, reviewed the species list for the Solomon Islands presented on Antwiki <http://www.antwiki.org/Solomon_Islands> , searched the Formis database (Porter and Wojcik 2012) for all relevant literature containing the term ‘Solomon’, and reviewed relevant taxonomic and regional literature. We also reviewed a dataset of ca. 1,040 specimen records of identified ants collected in the Solomon Islands that are deposited at the ANIC (Australian National Insect Collection, Canberra). We used the Bolton (2012) catalog to determine the valid names of all the species on the list. The Bolton (2012) catalog does not recognize the synonymy of *Cryptopone* with *Pachycondyla*, as implicitly proposed by Mackay and Mackay (2010), and the name is retained here as valid.

Names were eliminated where we found evidence of misidentification or geographic inconsistencies such as geographic names erroneously considered as belonging to the Solomon Islands. We also reconciled situations in which different authors may have referred to the same species by different valid names. For example, there were instances in which we believe one author referred to a taxon using its specific name, and another author referred to the same taxon by its infraspecific name. In cases such as these, and in the absence of additional evidence, we use the infraspecific name. We also note which other names we interpret as referring to the same taxon, and which publications those names occur in.

In addition to the valid names, we also use morphospecies codes to refer to presumptive species that either we or previous authors were unable to determine. The morphospecies code is ‘BP’ (The administrative code for the Solomon Islands) followed and a unique two-digit number (e.g. ‘*Camponotus* sp. BP01’).

Bougainville is considered to belong geographically but not politically to the Solomons. As such we do not include species recorded from Bougainville that have not also been reported from at least one of islands to its east.

### Survey of Makira

In addition to basing the present study on the aforementioned published records, we also include records from our own recent survey of the Solomons. Three of the authors (E.P.E., E.M.S., J.F.) collected ants in the Solomons from 30 January to 9 February, 2008. Aside from a few collections made on Mt. Austen (Guadalcanal I.), the survey primarily focused on Makira Island (formerly San Cristóbal) where we trekked and collected from Kirakira on the coast to the interior village of Maraone, reaching a maximum elevation of 912 m. Survey methods included hand collection and litter sifting along standardized transects using Winkler extraction bags. All specimens were collected into and stored in 95% ethanol. Pinned specimens were identified using the available literature and compared to type and determined material at the United States National Museum of Natural History (USNM), Washington D.C., USA, and the Museum of Comparative Zoology (MCZC), Cambridge, Massachusetts, USA. These two collections are the primary depositories for Mann’s type material and also include type material designated by W.L. Brown, W.M. Wheeler and E.O. Wilson. We include the species records from this survey with the literature records.

### Island records

Occurrence data of ant species on individual islands and island groups were compiled from the relevant literature. More detailed data with literature references for each species-island occurrence is available from the authors upon request. A map of the Solomon Islands (Figure 1) is also presented in which the name of every island and island group from which ant species have been recorded is labeled. The constituent islands comprising the listed island groups are presented in Table 1. In addition to including all taxa from Appendixes 1 and 2, we also include taxa from the 2008 survey of Makira that remain undetermined but might belong to previously described species. Inclusion of these additional taxa may weakly bias the observed species richness of Makira towards a higher value, but exclusion of these taxa would cause an even greater bias towards a lower value.

**Table 1.** Island groups and their constituent islands.<br/>

**Island Group**	**Islands**
Santa Cruz Is.	Anuta, Nendö (Santa Cruz), Nupani, Reef Is., Tikopia, Vanikoro
Olu Malau Is. (Three Sisters)	Malaupaina
Nggela Is. (Florida Is.)	Nggela Sule (Florida), Tulagi
New Georgia Is.	Kolombangarav, New Georgia, Rendova, Vangunu, Vella Lavella
Reef Is.	Matema

**Table 2.** Number of presumptive native species from Appendix 1 for each genus (arranged from greatest to least). Diverse genera with well-established subgenera are nested under the genus name and the species number of each is presented in parentheses.<br/>

**Genus (Subgenus)**	**Native spp.**	**%Total**
*Polyrhachis*	30	14
*Polyrhachis (Myrma)*	(7)	–
*Polyrhachis (Cyrtomyrma)*	(5)	–
*Polyrhachis (Chariomyrma)*	(4)	–
*Polyrhachis (Hedomyrma)*	(4)	–
*Polyrhachis (Myrmhopla)*	(3)	–
*Polyrhachis (Myrmatopa)*	(2)	–
*Polyrhachis (Myrmothrinax)*	(1)	–
*Polyrhachis (Hirtomyrma)*	(1)	–
*Pheidole*	15	7
*Camponotus*	14	7
*Camponotus (Colobopsis)*	(5)	–
*Tetramorium*	11	5
*Vollenhovia*	11	5
*Pachycondyla*	9	4
*Strumigenys*	9	4
*Crematogaster*	7	3
*Crematogaster (Crematogaster)*	(5)	–
*Crematogaster (Orthocrema)*	(2)	–
*Gnamptogenys*	6	3
*Cryptopone*	5	2
*Hypoponera*	5	2
*Myrmecina*	5	2
*Nylanderia*	5	2
*Ponera*	5	2
*Acropyga*	4	2
*Cerapachys*	4	2
*Eurhopalothrix*	4	2
*Leptogenys*	4	2
*Myopias*	4	2
*Odontomachus*	4	2
*Anochetus*	3	1
*Rogeria*	3	1
*Adelomyrmex*	2	1
*Arnoldius*	2	1
*Cardiocondyla*	2	1
*Carebara*	2	1
*Colobostruma*	2	1
*Iridomyrmex*	2	1
*Podomyrma*	2	1
*Prionopelta*	2	1
*Pristomyrmex*	2	1
*Proceratium*	2	1
*Rhytidoponera*	2	1
*Solenopsis*	2	1
*Stigmatomma*	2	1
*Turneria*	2	1
*Amblyopone*	1	<1
*Anonychomyrma*	1	<1
*Discothyrea*	1	<1
*Lordomyrma*	1	<1
*Monomorium*	1	<1
*Myopopone*	1	<1
*Oecophylla*	1	<1
*Opisthopsis*	1	<1
*Paraparatrechina*	1	<1
*Philidris*	1	<1
*Platythyrea*	1	<1
*Probolomyrmex*	1	<1
*Stereomyrmex*	1	<1
*Tapinoma*	1	<1
*Tetraponera*	1	<1

### Sampling analysis

We used our data compilation to estimate in a general sense how undersampled the Solomon Islands are for ants. First, we compared the species richness of individual islands in the Solomons with counts of the Fijian islands, which were the target of recent intensive sampling and taxonomic analysis (Sarnat and Economo 2012). We also compared the species richness of Makira from records before and after our 2008 survey.

## Results

### Ant records from the Solomon Islands

We present a list of nine subfamilies, 60 genera and 215 valid ant species and subspecies for the Solomon Islands based on our review of the literature and our recent collections from Makira (Appendix 1). We also present a list of 23 presumptively undescribed species that have also been recorded from the Solomons (Appendix 2). The generic composition and diversity of the Solomons is presented in Table 1. In total, our research suggests that the Solomon Islands support at least 237 unique ant taxa. The full species list with associated images and specimen data is available on Antweb.org <http://www.antweb.org/solomons.jsp> .

We excluded the following taxa from the list as they were reported from Bougainville but not from within the political boundaries of the Solomon Islands: *Cryptopone crassicornis* (Emery), *Polyrhachis aurea* (Mayr), *Polyrhachis obliqua* Stitz, and *Polyrhachis salomo* subsp. *hiram* Forel.

The following taxa were reported from the Solomon Islands, but are not believed to occur there either because the records were based on misidentified material or erroneous interpretation of locality data.

*Camponotus pallens* (Le Guillou, 1842): 316. Type locality: Tonga, Vavao. The website Antwiki.org, accessed 5 October 2012, listed this species under its Solomon Island webpage. The list was generated by extracting all species for which the Solomon Is. were listed as the type locality from the Bolton Catalog (Bolton et al. 2006). Although there are several Vavao islands in the Pacific (including in the Solomon Is.) the original description lists the type locality as *Vavao* (*íles des Amis*), which suggests Tonga (often referred to in older literature as the ‘Friendly Islands’) is the more likely country. Moreover, the species does not appear in any of the reviewed literature as occurring in the Solomons.

*Camponotus reticulatus* Roger, 1863: 139. Type locality: Sri Lanka. The first record of *Camponotus reticulatus* Roger appeared in Wilson (1962). Wilson explicitly applied *Camponotus reticulatus* Roger to the Solomons material that Wheeler (1934) referred to as *Camponotus reticulatus* subsp. *bedoti* Emery. In following the current classification (Bolton 2012), we accept *Camponotus bedoti* Emery as a valid species, and apply that name to all the material from the Solomons referred to as *Camponotus reticulatus* Roger. The decision to do so is somewhat arbitrary given the current state of taxonomy for Indo-Australian *Camponotus*, but we believe that both names refer to the same species in the Solomons.

*Hypoponera pallidula* (Emery, 1900): 320. Type locality: New Guinea. Mann (1919) reported this species as occurring in the Solomon Is., but Wilson (1958b) believed Mann’s specimens belonged to *Ponera sororcula* (= *Hypoponera sororcula*) Wilson.

*Leptogenys laeviceps* (Smith, 1857): 69. Type locality: Borneo. Mann (1919) reported this species as occurring in the Solomon Islands, but Wilson (1958a) considered Mann’s specimens to be a mixed series, part of which belong to *Leptogenys diminuta* Smith, F. and the other part to *Leptogenys oresbia* Wilson.

*Odontomachus haematodus* (Linnaeus, 1758): 582. Type locality: “America meridionali.” It is presumed that specimens referred to as *Odontomachus haematodus* by Mann (1919), Wheeler (1934; 1935a) and E. S. Brown (1959) prior to Wilson’s (1959b) revision belong instead to *Odontomachus simillimus* Smith, F.

*Odontomachus insularis* Guérin-Méneville, 1844: 423. Type locality: Cuba. Forel (1910) reported this species as occurring in the Solomon Is., but it is more likely that this was a misidentification and that the specimens he examined belong to *Odontomachus simillimus* Smith, F. *Odontomachus insularis* is not known from the Old World and was not included in Wilson (1959c).

*Pheidole punctulata* Mayr, 1866: 899. Type locality: South Africa. Forel (1910) reported this species as occurring in the Solomon Is., but it is more likely that the specimens he examined belong to the cosmopolitan tramp *Pheidole megacephala*.

*Philidris cordata* (Smith, F. 1859): 137. Type locality: Indonesia, Aru I. In his introduction, Greenslade (1972) treated *Iridomyrmex cordatus* (= *Philidris cordata*)Smith, F. as the senior synonym of *Iridomyrmex cordatus* var. *myrmecodiae* (= *Philidris myrmecodiae*) Emery. However, *Philidris myrmecodiae* has been accepted as a valid species since 1903 (Bolton 2012; Shattuck 1994). The correct name for the Solomons material would require comparison against type material for both taxa. In the meantime, our decision to use *Philidris myrmecodiae* rather than *Philidris cordata* reflects our belief that (1) insofar as the Solomon Is. are concerned, the use of both names refer to the same species; and (2) there is no taxonomic evidence proposed by Greenslade that Mann’s (1919) use of *Philidris myrmecodiae* was misapplied.

*Tetramorium obesum* André, 1887: 294. Type locality: India. Mann (1919) misidentified a series of specimens as belonging to *Tetramorium obesa* André that Bolton(1976) subsequently described as *Tetramorium vombis*.We assume here that the specimens referred to as *Tetramorium obesum* by Taylor (1976) are also *Tetramorium vombis*.

### Makira Island Survey

We collected a total of 67 described species and 30 presumptive species that are either undescribed or that we were unable to determine. Based on comparisons with type material, previously determined material and literature review, we suspect approximately 15 of the presumptive species are new to science. These taxa are included in Appendix 2. The survey added 67 new species records to Makira of taxa included in Appendixes 1 and 2, bringing the total number of species known from the island to 142. The survey also added 28 new species records to the Solomon Islands. Of these, six are previously described species (including three introduced species), and the remainder of species are included in Appendix 2.

### Island records and sampling analysis

Our research recovered species occurrence records for 32 individual islands and five island groups out of the approximately 75 named small to large individual islands and approximately 12 named island groups. These occurrence records are presented in Appendix 3. The 261 taxon names include the 215 described species and subspecies from Appendix 1, the 22 presumptive undescribed species from Appendix 2, and 24 additional morphospecies that likely represent a mixture of previously described species and undescribed species. This latter group is restricted to specimens collected during the 2008 Makira survey. The five islands with the highest number of species records, listed from greatest to least, are: Makira (142 spp.), Guadalcanal (107 spp.), Malaita (71 spp.), Santa Isabel (68 spp.), and Rennell (66 spp.). Fourteen individual islands have occurrence records for between 11–38 species. Thirteen individual islands have occurrence records for between 1–8 species.

The ten most widely distributed species, with the number of islands each is reported from, are: *Odontomachus simillimus* (27), *Anoplolepis gracilipes* (18), *Camponotus bedoti* (17), *Nylanderia vaga* (15), *Anochetus graeffei* (13), *Eurhopalothrix procera* (13), *Myopopone castanea* (13), *Oecophylla smaragdina subnitida* (13), *Pachycondyla stigma* (13), *Philidris myrmecodiae* (13). One hundred seven of the species and morphospecies included in Appendix 3 are only reported from single islands.

## Discussion

In total, our research suggests that the Solomon Islands support at least 237 unique ant species and subspecies. The poor sampling of many islands–some of which are quite large–and the unexamined material at the ANIC suggests that the true number is likely much greater. For example, our eight days of intensive hand collection and Winkler extractions on Makira added 67 new species records to the island (including all morphospecies) and 28 new records to the archipelago. Prior to the survey, Makira Island’s 75 species records were the second highest of the entire archipelago. Choiseul Island by comparison is approximately equal in area to Makira and closer to New Guinea, but the ant fauna of the island is virtually unknown with only eleven species recorded in the literature. There are approximately as many species known from the islands of Santa Isabel and Malaita as there are from Rennell, despite the substantially larger area of the former islands and their closer proximity to other large islands within the archipelago. The difference is that although no ant specialists have thoroughly sampled Rennell, general entomologists have collected there and the ant specimens of those surveys were the subject of several faunistic reviews (Taylor 1976; Wilson 1962). Besides Makira and Rennell Islands, the only island that has been moderately sampled–thanks to the works of Mann and Greenslade–is Guadalcanal.

**Figure 2.**
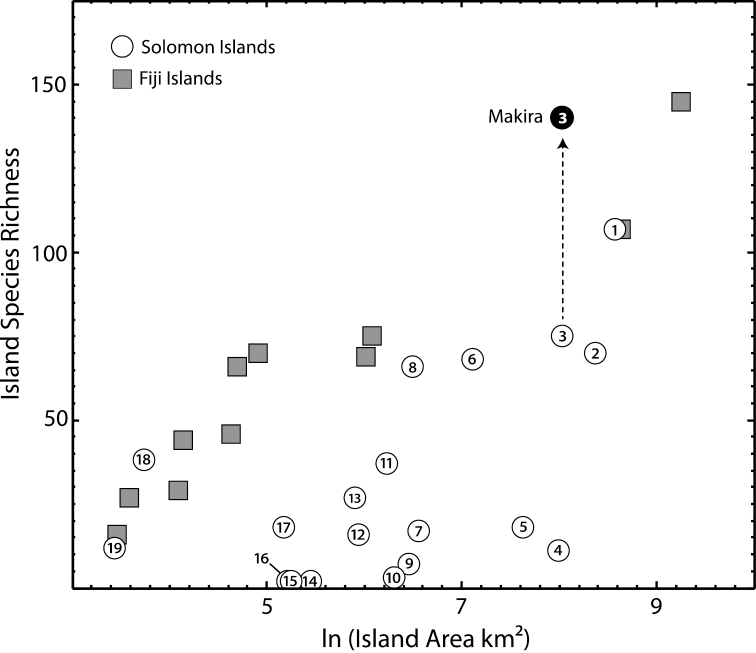
The relationship between islands area and known species richness. The figure presents individual islands in the Solomon (circles) and Fijian (squares) archipelagos, illustrating the undersampling of most Solomon Islands relative to the better collected Fiji Islands. For Makira, we present known species richness before (open circle) and after (filled circle) our recent collecting expedition. Numbers: **1** Guadalcanal **2** Malaita **3** Makira **4** Choiseul **5** New Georgia **6**Santa Isabel **7** Kolombangara **8** Rennell **9** Vella Lavella **10** Vangunu **11** Nendö (Santa Cruz) **12** Rendova **13** Nggela Sule **14** Shortland **15** Vanikoro **16** San Jorge **17** Russell Is. **18** Ugi **19** Savo.

Compared to Fijian islands of similar size, known species richness is generally much lower for individual islands within the Solomons, despite the fact that Fiji is much more isolated in the Pacific (Figure 2). This is likely due to relative sampling intensity of the two areas. Fiji has recently received intensive sampling efforts (Sarnat and Economo 2012), while richness differences among the Solomon Islands are still driven in large part by which islands were visited by W.M. Mann in 1916. For example, the 38 recorded species reported from the small island of Ugi (42 km^2^), where Mann resided and collected for several weeks, is a richness comparable with a similar-sized Fijian island. Several large islands not visited by Mann have almost no records (e.g. Choiseul 2,966 km^2^, 11 spp.; Kolombangara 704 km^2^, 17 spp.). Our modest survey of Makira, where we spent approximately one week of collecting time, increased known richness from 75 to 142 species. There is no doubt that such modest collecting efforts elsewhere in the archipelago would yield similar increases.

The species list compiled from our research suggests several interesting taxonomic patterns. For example, species richness across the 51 native ant genera of the Solomons appears uneven. The 30 *Polyrhachis* species represent 14% of the total native species. The nine most diverse genera (*Polyrhachis*, *Pheidole*, *Camponotus*, *Tetramorium*, *Vollenhovia*, *Pachycondyla*, *Strumigenys*, *Crematogaster*,and *Gnamptogenys*) collectively contain over half of the total native species, while fifteen genera are represented by a single native species.

Why is *Polyrhachis* so strongly represented in the Solomons? These results are likely biased to some extent by idiosyncratic collecting and taxonomic study. Besides the work of Mann, and to a lesser extent Greenslade, most of the collections from the Solomons have been made by more generalist collectors, which tend to take larger, more conspicuous ants that forage on and nest in vegetation–all of which are characteristic of *Polyrhachis*. Furthermore, Rudolf Kohout, who has access to the considerable collection of Solomons material at the ANIC, has devoted much of his taxonomic efforts towards revising the *Polyrhachis* of the Indo-Australian region (Kohout 1990; 1998; 2006; 2012). Despite these apparent biases, it is somewhat remarkable that with a single exception, the eight distinct *Polyrhachis* lineages that colonized the Solomons (as inferred from their subgeneric classifications) were unable to colonize, or at least persist in the more eastern Pacific islands. That single exception, *Polyrhachis rotumana* Wilson & Taylor,is known from the island of Rotuma which belongs politically to Fiji but is quite isolated from the Fijian archipelago and shares more geological and biological affinity with the islands of Polynesia.

*Pachycondyla* (9 native spp.), *Crematogaster* (7 native spp.)and *Gnamptogenys* (6 native spp.) are also among the most diverse ant genera in the Solomon Islands, but are either absent from or poorly represented in more easterly archipelagos. Fiji, for example, supports a single native *Gnamptogenys* species (*Gnamptogenys aterrima* Mann), and does not support any native *Pachycondyla* or *Crematogaster* species (Sarnat and Economo 2012). The Solomons are the known eastern limit for many ant genera. Out of the 51 genera native to the Solomons, the following 19 are not known to occur in the Pacific in or east of the Fijian archipelago: *Anonychomyrma*, *Arnoldius*, *Cardiocondyla*, *Colobostruma*, *Crematogaster*, *Cryptopone*, *Myopias*, *Myopopone*, *Myrmecina*, *Oecophylla*, *Opisthopsis*, *Pachycondyla*, *Podomyrma*, *Polyrhachis*, *Probolomyrmex*, *Rhytidoponera*, *Stereomyrmex*, *Tetraponera*, *Turneria*.

While additional sampling may prove otherwise, the current analysis of the Solomons ant fauna does not appear to support the type of *in situ* single-lineage radiations that characterize much of the Fijian ant fauna to the east. Parallels to the dramatic radiations of the *Pheidole roosevelti* group (Economo and Sarnat 2012; Sarnat 2008), *Lordomyrma* (Lucky and Sarnat 2008; Sarnat 2006), and the *Camponotus dentatus* group (Sarnat and Economo 2012) are largely unknown from the Solomons. It is likely that the Solomons ant fauna is derived more from relatively frequent colonization events from nearby New Guinea than from sweepstakes colonists that diversified into largely unoccupied ecological niches as occurred in the more isolated Fijian archipelago. Unlike New Guinea and Fiji,the Solomons do not support any endemic ant genera.

The importance of establishing baseline faunal inventories for the entire Solomon Island archipelago and its constituent islands is especially important when considering the growing environmental impacts resource extraction, plantation agriculture and invasive species are having on native biodiversity. Perhaps the greatest threat to native ant species in the Solomons is the spread of the Little Fire Ant (Fasi 2009). The introduction of *Wasmannia auropunctata* into the Solomon Islands is believed to have occurred around 1974, possibly with the arrival of coconut nurseries (Fabres and Brown 1978; Ikin 1984; Wetterer 1997). Foucaud et al. (2010) determined that a single clonal queen genotype is shared between the Melanesian populations of *Wasmannia auropunctata* from the Solomons, Vanuatu, Papua New Guinea and Australia, and suggested that the population spread by means of traditional exchange of plants and goods among Melanesian people. Although there have been reports of the ant’s effect on vertebrates in the Solomons, such as blinding dogs and attacking hatchlings of the ground-nesting Melanesian Scrubfowl (*Megapodius eremita* Hartlaub) (Wetterer 1997), and also its effect on food crops and subsistence agriculture (Fasi 2009), there have yet to be any studies examining the effect of *Wasmannia auropunctata* on native ant diversity in the Solomons. The potential for spread of *Wasmannia auropunctata* across the entire archipelago is high (Fasi 2009), and it is likely a matter of years before all the major islands are infested. We hope the research presented here will help facilitate more study of the neglected Solomon Island ant fauna and aid conservation efforts before *Wasmannia* and other environmental threats cause irrevocable harm.

## Figures and Tables

**Table:** 

**Taxon**	**Author**	**Year**	**Reference**
Amblyoponinae			
*Amblyopone australis*	Erichson, 1842: 261	1919	3, 8, 9, 13, 17, 38, 81
*Myopopone castanea*	(Smith, F. 1860): 105	1919	3, 8, 9, 13, 17, 21, 81
*Prionopelta majuscula*	Emery, 1897b: 595	2008	60, 81
*Prionopelta opaca*	Emery, 1897b: 596	1976	34, 60, 79, 81
*Stigmatomma celata*	(Mann, 1919): 279	1919	3, 8, 13, 17, 21, 34, 38, 81
*Stigmatomma gnoma*	Taylor, 1979: 829	1978	38, 81
Cerapachyinae			
*Cerapachys inconspicuus*	Emery, 1901: 153	1919	3, 8, 9, 18, 20, 34
*Cerapachys pawa*	Mann, 1919: 277	1919	3, 8, 20, 32
*Cerapachys terricola*	Mann, 1919: 277	1919	3, 8, 32, 79
Dolichoderinae			
*Anonychomyrma dimorpha*	(Viehmeyer, 1912): 7	1919	3, 8, 9, 79
*Arnoldius pusillus*	(Mayr, 1876): 83	1959	15
*Iridomyrmex anceps*	(Roger, 1863a): 164	1919	3, 6, 8, 15, 36, 81
*Iridomyrmex pallidus*	Forel, 1901: 22	1963	81
*Iridomyrmex rufoniger*	(Lowne, 1865): 279	1919	3, 8
Ochetellus glaber*	(Mayr, 1862): 705	2009	79
*Philidris myrmecodiae*	(Emery, 1887): 249	1919	3, 6, 8, 15, 16, 29^1^, 30^1^, 68^1^, 34^1^, 36^1^, 79
*Tapinoma (Micromyrma) indicum timidum*	Santschi, 1928	1959	15
Tapinoma melanocephalum*	(Fabricius, 1793): 353	1934	6, 8, 15, 18, 34, 36, 73, 81
*Tapinoma minutum*	Mayr, 1862: 703	1967	26
Technomyrmex albipes*	(Smith, F. 1861): 38	1910	1, 3, 8, 15, 18, 34, 36, 56, 79, 81
*Technomyrmex vitiensis*	Mann, 1921: 473	2008	79
*Turneria dahlii*	Forel, 1901: 17	1959	18, 34, 42, 81
*Turneria pacifica*	Mann, 1919: 361	1919	3, 42, 81
Ectatomminae			
*Gnamptogenys albiclava*	(Mann, 1919): 283	1919	3, 8, 11, 17, 54
*Gnamptogenys crenaticeps*	(Mann, 1919): 285	1919	3, 8, 11, 17, 54, 79
*Gnamptogenys lucida*	(Mann, 1919): 285	1919	3, 8, 11, 17, 54
*Gnamptogenys malaensis*	(Mann, 1919): 281	1919	3, 8, 11, 17, 54, 79
*Gnamptogenys preciosa*	Lattke, 2004: 66	2004	54, 81
*Gnamptogenys solomonensis*	Lattke, 2004: 66	2004	54, 81
*Rhytidoponera araneoides*	(Le Guillou, 1842): 317	1910	1, 3, 14, 17, 79, 81
*Rhytidoponera chalybaea*	Emery, 1901b: 51	1959	15
Formicinae			
*Acropyga acutiventris*	Roger, 1862: 243	1919	3, 8, 53, 79, 81
*Acropyga lauta*	Mann, 1919: 365	1919	3, 8, 53, 79, 81
*Acropyga oceanica*	Emery, 1900: 333	2008	79
*Acropyga pallida*	(Donisthorpe, 1938): 598	1965	81
Anoplolepis gracilipes*	Smith, F. 1857: 55	1919	3, 6, 8, 15, 15, 18, 29, 30, 68, 34, 36, 81
Brachymyrmex obscurior*	Forel, 1893: 345	1976	34, 79
*Camponotus (Myrmamblys) bedoti*	Emery, 1893: 196	1919	3, 6, 8, 15, 18^2^, 34^2^, 36^2^
*Camponotus chloroticus*	Emery, 1897b: 574	1959	15
*Camponotus elysii*	Mann, 1919: 372	1919	3, 8
*Camponotus guppyi*	Mann, 1919: 370	1919	3, 8
*Camponotus loa*	Mann, 1919: 373	1919	3, 8
*Camponotus loa belli*	Mann, 1919: 375	1919	3, 8
*Camponotus novaehollandiae*	Mayr, 1870: 939	1919	3
Nylanderia bourbonica*	(Forel, 1886): 210	1959	15, 34, 36, 81
*Nylanderia braueri glabrior*	(Forel, 1902): 490	1954	81
*Nylanderia dichroa*	Wheeler 1934: 181	1934	6, 8, 81
*Nylanderia manni*	Donisthorpe, 1941: 41	1941	72, 15, 36
*Nylanderia obscura bismarckensis*	(Forel, 1901): 26	1919	3, 6, 8
*Nylanderia stigmatica*	Mann, 1919: 367	1919	3, 8, 62, 79, 81
Nylanderia vaga*	(Forel, 1901): 26	1934	6, 8, 18, 26, 34, 36, 79, 81
Nylanderia vividula*	(Nylander, 1846): 900	1919	3, 15, 79
*Oecophylla smaragdina subnitida*	Emery 1892: 565	1910	1, 3, 6, 8, 15^3^, 16^3^, 29^3^, 30^3^, 68^3^, 36^3^, 79^3^, 81^3^
*Opisthopsis manni*	Wheeler, W.M. 1918: 361	1918	2, 3, 8, 15
*Paraparatrechina minutula*	(Forel, 1901): 25	1919	3, 8, 15, 34, 79, 81
Paratrechina longicornis*	(Latreille, 1802): 113	1919	3, 8, 15, 34, 79, 81
Plagiolepis alluaudi*	Emery, 1894: 71	1959	15
*Polyrhachis (Myrma) andromache*	Roger, 1863b: 8	1959	3^4^, 18, 34^5^, 79
*Polyrhachis (Hedomyrma) annae*	Mann, 1919: 377	1919	3, 6, 8, 15, 18, 34
*Polyrhachis (Chariomyrma) arcuata acutinota*	Forel, 1901: 31	1934	6
*Polyrhachis (Hedomyrma) campbelli*	Mann, 1919: 376	1919	3, 8, 79
*Polyrhachis (Myrmothrinax) dahlii*	Forel, 1901: 30	1919	3, 8, 9, 64
*Polyrhachis (Cyrtomyrma) emeryana*	Mann, 1919: 390	1919	3, 8, 55
*Polyrhachis (Cyrtomyrma) fulakora*	Mann, 1919: 389	1919	3, 8, 15, 55
*Polyrhachis (Hedomyrma) geminata*	Mann, 1919: 376	1919	3, 8, 79
*Polyrhachis greensladei*	Kohout, 1990: 503	1990	74
*Polyrhachis (Myrma) ithona*	Smith, F., 1860: 99	1934	6, 8
*Polyrhachis (Cyrtomyrma) johnsoni*	Mann, 1919: 390	1919	3, 8, 55
*Polyrhachis (Chariomyrma) kaipi*	Mann, 1919: 382	1919	3, 6, 8, 79
*Polyrhachis (Myrma) labella brunneipes*	Wheeler, 1934	1934	6, 8
*Polyrhachis (Myrma) litigiosa*	Emery, 1897b: 581	1919	3, 8, 79
*Polyrhachis (Myrma) malaensis*	Mann, 1919: 386	1919	3, 8
*Polyrhachis nofra*	Bolton, 1975: 9	1975	31
*Polyrhachis (Myrmatopa) osae*	Mann, 1919: 384	1919	3, 6, 8, 9, 15
*Polyrhachis pacifica*	Kohout, 2006: 140	2006	55
*Polyrhachis (Chariomyrma) rere*	Mann, 1919: 381	1919	3, 6, 8, 15
*Polyrhachis (Myrmhopla) saevissima argentea*	Mayr, 1862: 82	1919	3, 8, 9
*Polyrhachis (Myrma) salomo*	Forel, 1910: 87	1910	1, 3, 8, 15
*Polyrhachis (Hedomyrma) santschii*	Mann, 1919: 375	1919	3, 8
*Polyrhachis setosa*	Kohout, 2006: 141	2006	55
*Polyrhachis (Myrma) similis*	Viehmeyer, 1912: 8	1919	3, 8
*Polyrhachis (Cyrtomyrma) ugiensis*	Mann, 1919: 389	1919	3, 8, 55, 79
*Polyrhachis (Myrmatopa) ulysses*	Forel, 1910: 91	1910	1, 3, 8
*Polyrhachis (Cyrtomyrma) undulata*	Kohout, 2006: 142	2006	55, 79
*Polyrhachis (Myrmhopla) wheeleri*	Mann, 1919: 387	1919	3, 8, 9
Myrmicinae			
Cardiocondyla kagutsuchi*	Terayama, 1999: 100	2009	79
*Cardiocondyla nivalis*	Mann, 1919: 317	1919	3, 8, 34, 36
*Cardiocondyla nuda*	(Mayr, 1866): 508	1959	15, 34, 36, 51, 59
*Carebara atoma*	(Emery, 1900): 328	1919	3, 8, 34, 36, 79
*Carebara viehmeyeri*	(Mann, 1919): 331	1919	3, 8, 79
*Colobostruma foliacea*	Emery, 1897a: 573	2000	48, 81
*Crematogaster (Crematogaster) abrupta*	Mann, 1919: 320	1935	8, 15, 61
*Crematogaster (Crematogaster) elysii*	Mann, 1919: 319	1935	8, 3, 61
*Crematogaster (Crematogaster) foxi*	Mann, 1919: 321	1935	8, 3, 61
*Crematogaster (Crematogaster) nesiotis*	Mann, 1919: 322	1935	8, 3, 61
*Crematogaster (Crematogaster) obnigra*	Mann, 1919: 323	1919	3, 15, 61
*Crematogaster (Orthocrema) scita*	Forel, 1902: 409	1959	15
*Crematogaster (Orthocrema) wheeleri*	Mann, 1919: 318	1935	8, 3, 61
*Eurhopalothrix brevicornis*	(Emery, 1897a): 572	1977	36, 80, 28, 82
*Eurhopalothrix greensladei*	Taylor, 1968: 342	1968	28, 82
*Eurhopalothrix isabellae*	(Mann, 1919): 357	1919	3, 8, 22, 80, 28, 82
*Eurhopalothrix procera*	(Emery, 1897a): 572	1919	3, 8, 22, 28, 79, 81, 82
*Lordomyrma epinotalis*	(Mann, 1919): 343	1919	3, 8, 34, 58, 79
*Monomorium australicum*	Forel, 1907:20	1919	3, 8, 15, 34, 36
Monomorium destructor*	(Jerdon, 1851): 105	1959	18, 34
Monomorium floricola*	(Jerdon, 1851): 107	1959	15, 34, 36, 41, 79, 81
Monomorium pharaonis*	(Linnaeus, 1758): 580	1919	3, 8, 15, 34, 41, 81
*Myrmecina modesta*	Mann, 1919: 335	1919	3, 8, 34^6^
*Myrmecina modesta subarmata*	Mann, 1919: 337	1919	3, 8
*Myrmecina transversa*	Emery, 1897a: 582	2008	79
*Pheidole belli*	Mann, 1919: 306	1919	3, 8
*Pheidole erato*	Mann, 1919: 307	1919	3, 8
*Pheidole fuscula*	Emery, 1900: 325	1919	3, 8
*Pheidole isis*	Mann, 1919: 311	1919	3, 8
*Pheidole isis taki*	Mann, 1919: 314	1919	3, 8, 79
Pheidole megacephala*	(Fabricius, 1793): 361	1910	1^7^, 6, 8, 15, 26, 30, 34, 81
*Pheidole mendanai*	Mann, 1919: 311	1919	3, 8
*Pheidole nindi*	Mann, 1919: 314	1919	3, 8, 34, 36, 79
*Pheidole oceanica*	Mayr, 1866: 510	1919	3, 8, 15, 18, 34, 36, 79
*Pheidole philemon*	Forel, 1910: 44	1910	1, 3, 8, 15, 79
*Pheidole sexspinosa*	Mayr, 1870: 977	1919	3, 8, 34, 36, 79
*Pheidole sexspinosa fuscescens*	Emery, 1900: 323	1919	3, 8, 18
*Pheidole umbonata*	Mayr, 1870: 978	1919	3, 8, 15, 18, 34, 36
*Podomyrma basalis salomo*	Mann, 1919: 333	1919	3, 8
*Podomyrma basalis woodfordi*	Mann, 1919: 334	1919	3, 8
*Pristomyrmex levigatus*	Emery, 1897a: 583	1919	3, 52, 79
*Pristomyrmex obesus*	Mann, 1919: 339	1919	3, 8, 80, 52
*Rogeria megastigmatica*	Kugler, C. 1994: 35	1994	45, 79
*Rogeria stigmatica*	Emery, 1897: 589	1919	3, 8, 34, 45
*Romblonella elysii*	(Mann, 1919): 346	1919	3, 8, 44
Solenopsis geminata*	(Fabricius, 1804): 423	1977	36
*Solenopsis papuana*	Emery, 1900: 330	1919	3, 79
*Solenopsis pawaensis*	Mann, 1919: 329	1919	3, 79
*Stereomyrmex dispar*	(Wheeler, W.M. 1934): 175	1934	6, 18, 34, 44
*Strumigenys chyzeri*	Emery, 1897a: 576	1919	3, 48, 79
*Strumigenys decollata*	Mann, 1919: 353	1919	3, 8, 12, 48
Strumigenys emmae*	Emery, 1890: 70	1976	34, 36, 48, 81
*Strumigenys eurycera*	Emery, 1897a: 581	2000	48, 81
*Strumigenys frivaldszkyi*	Emery, 1897: 580	1976	34, 48, 79
Strumigenys godeffroyi*	Mayr, 1866: 516	1919	3, 15, 34, 36, 47, 48, 79
*Strumigenys karawajewi*	(Brown, 1948): 44	1976	34, 46, 48, 79, 81
Strumigenys membranifera*	(Emery, 1869): 24	2000	48, 36, 81
*Strumigenys mocsaryi*	(Emery, 1897a): 580	2000	48
Strumigenys rogeri*	Emery, 1890: 68	2000	48
*Strumigenys szalayi*	Emery, 1897: 578	2000	48, 79
*Strumigenys undras*	Bolton, 2000: 752	2000	48
*Strumigenys yaleopleura*	Brown, 1988: 41	2000	48
*Tetramorium antennatum*	(Mann, 1919): 350	1919	3
*Tetramorium aspersum*	(Smith, F. 1865): 72	1919	3, 6, 8, 35, 79
Tetramorium bicarinatum*	(Nylander, 1846): 1061	1919	3^8^, 6^8^, 8^8^, 15^8^, 34^8^, 35, 36
*Tetramorium carinatum*	(Smith, F. 1859): 148	1919	3, 8
*Tetramorium insolens*	(Smith, F., 1861)	1934	6, 8, 18, 34, 35
Tetramorium lanuginosum*	Mayr, 1870: 976	1935	8, 69
*Tetramorium mayri*	(Mann, 1919: 351)	1919	3, 8, 79
*Tetramorium melanogyna*	Mann, 1919: 345	1919	3, 8, 79
*Tetramorium mutatum*	Bolton, 1985: 247	1919	3, 8, 69
*Tetramorium pacificum*	Mayr, 1870: 976	1934	6, 8, 18, 34, 35
*Tetramorium salomo*	Mann, 1919: 344	1935	8, 35, 79
Tetramorium simillimum*	(Smith, F. 1851): 118	1959	15, 34, 35, 36, 79
*Tetramorium tonganum*	Mayr, 1870: 976	1919	3, 8, 15, 18, 34, 35
*Tetramorium vombis*	Bolton, 1976: 358	1985	3^9^, 34^9^, 69
*Vollenhovia dentata*	Mann, 1919: 325	1919	3, 8, 24, 79
*Vollenhovia dentata marginata*	Mann, 1919: 327	1919	3, 8, 24
*Vollenhovia elysii*	Mann, 1919: 327	1919	3, 8, 24
*Vollenhovia foveaceps*	Mann, 1919: 328	1919	3, 8, 24
*Vollenhovia loboii*	Mann, 1919: 324	1919	3, 8, 24
*Vollenhovia oblonga*	(Smith, F. 1861): 46	1959	18, 34, 43
*Vollenhovia oblonga pedestris*	(Smith, F. 1860): 107	1919	3, 8, 15, 79
*Vollenhovia subtilis*	Emery, 1887: 454	1919	3, 8
Wasmannia auropunctata*	(Roger, 1863a): 183	1984	40, 75, 76, 77, 79
Ponerinae			
*Anochetus cato*	Forel, 1901: 6	1919	3, 8, 17, 19, 79, 81
*Anochetus graeffei*	Mayr, 1870: 961	1919	3, 8, 15, 17, 19, 34, 36, 65, 79, 81
*Anochetus isolatus*	Mann, 1919: 302	1919	3, 8, 17, 19, 34, 37, 65, 79, 81
*Cryptopone butteli*	Forel, 1913: 9	1965	81
*Cryptopone crassicornis*	(Emery, 1897): 533	1965	81
*Cryptopone fusciceps*	(Emery, 1900): 321	1919	3, 4, 8, 14, 17, 81
*Cryptopone testacea*	(Emery, 1893): cclxxv	1919	3, 4, 8, 14, 17, 32, 81
*Hypoponera biroi*	(Emery, 1900): 7	1959	17, 34
*Hypoponera confinis*	(Roger, 1860): 284	1959	17
*Hypoponera pallidula*	(Emery, 1900): 320	1919	3, 8, 9
*Hypoponera papuana*	(Emery, 1900): 319	1919	3, 8, 79
*Hypoponera pruinosa*	(Emery, 1900): 319	1919	3, 8, 9, 14, 17, 34, 79
Hypoponera punctatissima*	(Roger, 1859): 246	1976	34, 79
Hypoponera ragusai*	(Forel, 1899): 28	1919	3, 8, 14, 17, 36
*Hypoponera sororcula*	(Wilson, 1958a): 338	1958	14, 17
*Leptogenys diminuta*	(Smith, F. 1857): 69	1919	3, 8, 17, 79
*Leptogenys foreli*	Mann, 1919: 297	1919	3, 8, 13, 17, 18^10^, 34^10^
*Leptogenys oresbia*	Wilson, 1958b: 131	1958	3^11^, 13, 17
*Leptogenys truncata*	Mann, 1919: 26	1919	3, 17
*Odontomachus malignus*	Smith, F. 1859: 144	1919	3, 17, 18^12^, 19, 33, 34^12^, 63, 81
*Odontomachus rufithorax*	Emery, 1911: 534	1919	3, 17, 19, 33, 81
*Odontomachus saevissimus*	(Smith, F. 1858)	1959	15, 33, 81
*Odontomachus simillimus*	(Smith, F. 1858): 80	1910	1^13^, 3^14^, 6^14^, 8^14^, 15^14^, 17, 18, 19, 26, 34, 36, 79, 81
*Pachycondyla acuta*	Emery, 1900	1958	14, 17
*Pachycondyla aequalis*	(Mann, 1919): 289	1919	3, 8, 14, 17, 79
*Pachycondyla croceicornis*	(Emery, 1900): 315	1919	3, 14, 17, 36, 79
*Pachycondyla darwinii*	(Forel, 1893): 460	1959	17
*Pachycondyla exarata*	Emery, 1901b: 156	1919	3, 8
*Pachycondyla manni*	(Viehmeyer, 1924): 228	1924	71, 14, 17
*Pachycondyla melancholica*	Smith, F. 1865: 71	1919	3
*Pachycondyla papuana*	(Viehmeyer, 1914): 608	1919	3, 9
*Pachycondyla sheldoni*	(Mann, 1919): 292	1919	3, 8, 14, 17
Pachycondyla stigma*	(Fabricius, 1804): 400	1919	3, 8, 9, 15, 17, 18, 34, 79
*Platythyrea parallela*	(Smith, F., 1859): 143	1919	3, 9, 17
*Ponera clavicornis*	Emery, 1900: 317	1919	3, 8, 10, 25, 34^15^, 81
*Ponera incerta*	(Wheeler, W.M. 1933): 18	1959	17, 25, 81
*Ponera swezeyi*	(Wheeler, W.M. 1933): 16	2009	79
*Ponera szaboi*	Wilson, 1957: 371	1976	34
*Ponera tenuis*	(Emery, 1900): 321	1965	81
Proceratiinae			
*Discothyrea clavicornis*	Emery, 1897b: 593	1919	3, 8, 9, 17, 81
*Probolomyrmex salomonis*	Taylor, 1965: 358	1965	23, 66, 81
*Proceratium austronesicum*	De Andrade, in Baroni Urbani and De Andrade 2003: 313	2003	50, 81
*Proceratium papuanum*	Emery, 1897b: 592	2003	50, 81
Pseudomyrmecinae			
*Tetraponera laeviceps*	(Smith, F. 1859): 145	1919	3, 8, 49

**Table:** 

**Taxon**	**Notes**	**Year**	**Reference**
*Adelomyrmex* sp. BP02	nr. *hirsutus*	2008	79
*Adelomyrmex* sp. BP03	as “*Adelomyrmex (Arctomyrmex)* sp.”	1976	34
*Arnoldius* sp. BP01	as “nr. *flavus*”	1959	15
*Camponotus* sp. BP02	nr. *guppyi*	2008	79
*Camponotus* sp. BP05	nr. *elysii*	2008	79
*Camponotus* sp. BP06	as “*Camponotus (Colobopsis)* sp. A”	1976	34
*Camponotus* sp. BP07	as “*Camponotus (Colobopsis)* sp. B”	1976	34
*Camponotus* sp. BP08	as “*Camponotus (Colobopsis)* sp. C”	1976	34
*Camponotus* sp. BP09	as “*Camponotus (Colobopsis)* spp. (2)”	1959	18,34
*Camponotus* sp. BP10	as “*Camponotus (Colobopsis)* spp. (2)”	1959	18,34
*Cerapachys* sp. BP01	as “*Cerapachys*? (*Syscia*) sp. 1”	1959	18,34
*Colobostruma* sp. BP01	nr. *foliacea*	2008	79
*Cryptopone* sp. BP01	nr. *testacea*	2008	79
*Myopias* sp. BP01		2008	79
*Myopias* sp. BP02		2008	79
*Myopias* sp. BP03		2008	79
*Myopias* sp. BP04	as “*Myopias* cf. *tenuis*”	1983	39
*Myrmecina* sp. BP01		2008	79
*Myrmecina* sp. BP03		2008	79
*Pheidole* sp. BP02		2008	79
*Pheidole* sp. BP12	nr. *mendanai*	2008	79
*Pheidole* sp. BP13	as “*Pheidole (Pheidolacanthinus)* sp.”	1976	34
*Platythyrea* sp. BP01	as “*Platythyrea* sp.”	1976	34
*Polyrhachis* sp. BP01	*Polyrhachis (Myrmhopla) *nr.* bismarckensis *	2008	79
*Polyrhachis* sp. BP03	as “*Polyrhachis (Chariomyrma)* sp.”	1976	34
*Rogeria* sp. BP01	nr. *stigmatica*	2008	79
*Strumigenys* sp. BP05	nr. *mocsaryi*	2008	79
*Vollenhovia* sp. BP01	nr. *elysii*	2008	79
*Vollenhovia* sp. BP02	nr. *loboii*	2008	79
*Vollenhovia* sp. BP03	as “*Vollenhovia* sp.”	1976	34

**Table:** 

*Taxon*	*Anuta*	*Bellona*	*Choiseul*	*Guadalcanal*	*Kolombangara*	*Makira*	*Malaita*	*Malaupaina*	*Matema*	*Mbanika*	*New Georgia*	***NEW GEORGIA IS*.**	***NGGELA IS*.**	*Nggela Sule*	*Nupani*	***OLU MALAU IS*.**	*Ontong Java Is*.	*Owaraha*	***REEF IS*.**	*Rendova*	*Rennell*	*Russell Is*.	*San Jorge*	*Santa Catalina*	*Santa Cruz*	***SANTA CRUZ IS*.**	*Santa Isabel*	*Savo*	*Shortland*	*Sikaiana*	*Tikopia*	*Tulagi*	*Ugi*	*Vangunu*	*Vanikoro*	*Vella Lavella*	*SOLOMON IS*.	***Total***
**Acropyga**																																						
*Acropyga acutiventris*				x		x	x					x								x							x						x					7
*Acropyga lauta*				x	x	x	x					x	x														x											7
*Acropyga oceanica*						x																																1
*Acropyga pallida*					x																																	1
**Adelomyrmex**																																						
*Adelomyrmex* sp. BP02*						x																																1
*Adelomyrmex* sp. BP03*		x																			x																	2
**Amblyopone**																																						
*Amblyopone australis*				x			x					x																									x	3
**Anochetus**																																						
*Anochetus cato*						x	x	x				x	x	x		x				x							x					x	x				x	11
*Anochetus graeffei*		x		x		x	x				x	x	x	x							x				x	x	x					x					x	13
*Anochetus isolatus*			x	x		x		x								x					x				x	x											x	8
**Anonychomyrma**																																						
*Anonychomyrma dimorpha*						x	x																				x										x	3
**Anoplolepis**																																						
*Anoplolepis gracilipes*		x	x	x	x	x	x		x			x	x		x				x		x	x		x	x	x	x	x										18
**Arnoldius**																																						
*Arnoldius* sp. BP01				x																																		1
*Arnoldius pusillus*				x																																		1
**Brachymyrmex**																																						0
*Brachymyrmex obscurior*		x				x															x																	3
**Camponotus**																																						
*Camponotus bedoti*	x	x		x			x				x	x	x	x	x			x		x	x	x		x	x	x						x						17
*Camponotus chloroticus*				x														x																				2
*Camponotus elysii*						x		x								x																						3
*Camponotus guppyi*							x																															1
*Camponotus loa*						x	x	x					x			x		x							x	x						x	x					10
*Camponotus loa belli*													x	x																								2
*Camponotus novaehollandiae*						x																			x	x	x											4
*Camponotus* sp. BP01						x																																1
*Camponotus* sp. BP02*						x																																1
*Camponotus* sp. BP04						x																																1
*Camponotus* sp. BP05*						x																																1
*Camponotus* sp. BP06*																					x																	1
*Camponotus* sp. BP07*																					x																	1
*Camponotus* sp. BP08*		x																			x																	2
*Camponotus* sp. BP09*																					x																	1
*Camponotus* sp. BP10*																					x																	1
**Cardiocondyla**																																						
*Cardiocondyla kagutsuchi*						x																																1
*Cardiocondyla nivalis*				x		x				x		x								x	x							x								x		8
*Cardiocondyla nuda*				x			x														x			x			x											5
**Carebara**																																						
*Carebara atoma*		x		x		x															x						x											5
*Carebara viehmeyeri*						x																																1
**Cerapachys**																																						
*Cerapachys inconspicuus*																					x						x										x	2
*Cerapachys pawa*																																	x				x	1
*Cerapachys terricola*						x							x	x		x											x					x					x	6
*Cerapachys* sp. BP01*																					x																	1
**Colobostruma**																																						
*Colobostruma foliacea*			x	x								x								x		x					x											6
*Colobostruma* sp. BP01*						x																																1
**Crematogaster**																																						
*Crematogaster abrupta*				x				x								x																						3
*Crematogaster elysii*								x								x																						2
*Crematogaster foxi*						x																																1
*Crematogaster nesiotis*																						x																1
*Crematogaster obnigra*				x																		x																2
*Crematogaster scita*				x																																		1
*Crematogaster wheeleri*							x																															1
**Cryptopone**																																						
*Cryptopone butteli*				x																																		1
*Cryptopone crassicornis*				x																																		1
*Cryptopone fusciceps*				x		x	x					x															x										x	5
*Cryptopone testacea*		x		x	x	x						x									x						x						x				x	8
*Cryptopone* sp. BP01*						x																																1
**Dilobocondyla**																																						
*Dilobocondyla* sp. BP01																					x																	1
**Discothyrea**																																						
*Discothyrea clavicornis*				x		x	x													x		x	x				x		x								x	8
**Eurhopalothrix**																																						
*Eurhopalothrix brevicornis*				x								x	x	x													x									x		5
*Eurhopalothrix greensladei*				x																																		1
*Eurhopalothrix isabellae*				x								x															x									x		4
*Eurhopalothrix procera*				x		x	x	x				x	x	x		x				x					x	x	x	x				x						14
**Gnamptogenys**																																						
*Gnamptogenys albiclava*				x																							x										x	2
*Gnamptogenys crenaticeps*						x																					x										x	2
*Gnamptogenys lucida*							x																														x	1
*Gnamptogenys malaensis*					x	x	x				x	x										x												x		x	x	8
*Gnamptogenys preciosa*				x																																		1
*Gnamptogenys solomonensis*				x	x							x																										3
*Gnamptogenys* sp. BP03						x																																1
**Hypoponera**																																						
*Hypoponera biroi*																					x																x	1
*Hypoponera confinis*																																					x	0
*Hypoponera pallidula*							x																				x										x	2
*Hypoponera papuana*						x		x								x											x						x				x	5
*Hypoponera pruinosa*		x				x	x	x					x	x		x					x						x					x	x				x	11
*Hypoponera punctatissima*						x															x																	2
*Hypoponera ragusai*				x		x																															x	2
*Hypoponera sororcula*																																					x	0
*Hypoponera* sp. BP01						x																																1
*Hypoponera* sp. BP06						x																																1
*Hypoponera* sp. BP07						x																																1
*Hypoponera* sp. BP08						x																																1
**Iridomyrmex**																																						
*Iridomyrmex anceps*				x	x		x		x		x	x					x		x			x					x	x										11
*Iridomyrmex pallidus*				x																																		1
*Iridomyrmex rufoniger*							x																															1
**Leptogenys**																																						
*Leptogenys diminuta*						x		x								x																	x				x	4
*Leptogenys foreli*							x	x								x					x																x	4
*Leptogenys oresbia*								x																													x	1
*Leptogenys truncata*																									x	x											x	2
**Lordomyrma**																																						
*Lordomyrma epinotalis*						x	x			x			x								x						x						x					7
**Monomorium**																																						
*Monomorium australicum*		x		x		x															x												x					5
*Monomorium destructor*																					x																	1
*Monomorium floricola*		x		x		x					x	x	x								x											x						8
*Monomorium pharaonis*				x			x									x					x												x					5
**Myopias**																																						
*Myopias* sp. BP01*						x																																1
*Myopias* sp. BP02*						x																																1
*Myopias* sp. BP03*						x																																1
*Myopias* sp. BP04*				x		x																																2
**Myopopone**																																						
*Myopopone castanea*				x	x	x	x				x	x	x	x											x	x	x					x			x		x	13
**Myrmecina**																																						
*Myrmecina modesta*						x		x								x					x				x	x	x						x					8
*Myrmecina subarmata*							x																															1
**Myrmecina**																																						
*Myrmecina transversa*						x																																1
*Myrmecina* sp. BP01*						x																																1
*Myrmecina* sp. BP03*						x																																1
**Nylanderia**																																						
*Nylanderia bourbonica*		x		x																	x																	3
*Nylanderia braueri glabrior*				x		x															x							x										4
*Nylanderia dichroa*			x	x	x	x	x					x	x							x								x										9
*Nylanderia manni*				x																																		1
*Nylanderia bismarckensis*						x															x						x											3
*Nylanderia stigmatica*				x	x	x	x	x								x																	x					7
*Nylanderia vaga*	x	x		x	x	x			x						x				x		x			x		x	x	x		x			x					15
*Nylanderia vividula*				x		x																											x					3
**Ochetellus**																																						
*Ochetellus glaber*						x																																1
**Odontomachus**																																						
*Odontomachus malignus*				x			x														x				x	x	x										x	6
*Odontomachus rufithorax*			x										x	x													x										x	4
*Odontomachus saevissimus*				x	x																																x	2
*Odontomachus simillimus*	x	x	x	x	x	x	x	x	x			x	x	x	x	x	x		x		x	x			x	x	x	x			x	x	x	x	x		x	27
**Oecophylla**																																						
*Oecophylla subnitida*				x		x	x	x					x	x		x		x				x					x	x				x	x					13
**Opisthopsis**																																						0
*Opisthopsis manni*				x		x	x	x								x						x																6
**Pachycondyla**																																						
*Pachycondyla acuta*																x																					x	1
*Pachycondyla aequalis*						x	x						x	x																							x	4
*Pachycondyla croceicornis*				x		x																					x										x	3
*Pachycondyla darwinii*																																					x	0
*Pachycondyla exarata*													x														x					x						3
*Pachycondyla manni*						x																					x										x	2
*Pachycondyla melancholica *																									x													1
*Pachycondyla papuana*						x																					x										x	2
*Pachycondyla sheldoni*						x																															x	1
*Pachycondyla stigma*				x		x	x				x	x	x	x							x				x	x	x					x	x				x	13
*Pachycondyla* sp. BP01						x																																1
**Paraparatrechina**																																						
*Paraparatrechina minutula*		x		x		x															x						x						x					6
**Paratrechina**																																						
*Paratrechina longicornis*		x	x	x		x	x					x						x			x				x	x												10
**Pheidole**																																						
*Pheidole belli*							x																															1
*Pheidole erato*																											x											1
*Pheidole fuscula*																											x											1
*Pheidole isis*																											x											1
*Pheidole taki*						x																																1
*Pheidole megacephala*	x			x			x		x								x		x		x					x											x	8
*Pheidole mendanai*																											x											1
*Pheidole nindi*				x		x															x												x					4
*Pheidole oceanica*		x		x		x	x	x								x					x				x	x	x						x					11
*Pheidole philemon*				x		x	x	x								x									x	x											x	7
*Pheidole sexspinosa*		x		x		x	x						x								x				x	x	x					x						10
*Pheidole fuscescens*													x	x																								2
*Pheidole umbonata*		x		x		x	x														x												x					6
*Pheidole* sp. BP02*						x																																1
*Pheidole* sp. BP09						x																																1
*Pheidole* sp. BP10						x																																1
*Pheidole* sp. BP11						x																																1
*Pheidole* sp. BP12*						x																																1
*Pheidole* sp. BP13*																					x																	1
**Philidris**																																						
*Philidris myrmecodiae*		x		x		x	x						x	x		x		x			x				x	x	x						x					13
**Plagiolepis**																																						
*Plagiolepis alluaudi*				x																																		1
**Platythyrea**																																						
*Platythyrea parallela*																									x	x											x	2
*Platythyrea* sp. BP01*																					x																	1
**Podomyrma**																																						
*Podomyrma salomo*							x						x	x																		x						4
*Podomyrma woodfordi*								x								x																						2
**Polyrhachis**																																						
*Polyrhachis andromache*						x																																1
*Polyrhachis annae*		x		x		x		x	x							x			x		x				x	x												10
*Polyrhachis acutinota*				x																																		1
*Polyrhachis argentea*							x						x	x																							x	3
*Polyrhachis brunneipes*		x					x																															1
*Polyrhachis campbelli*						x																x																2
*Polyrhachis dahlii*				x		x	x	x					x	x		x																x					x	8
*Polyrhachis emeryana*							x																															1
*Polyrhachis fulakora*				x																							x											2
*Polyrhachis geminata*						x							x	x																								3
*Polyrhachis greensladei*				x																								x										2
*Polyrhachis ithona*									x										x																			2
*Polyrhachis johnsoni*								x				x				x				x																		4
*Polyrhachis kaipi*	x					x	x																			x												4
*Polyrhachis litigiosa*						x																					x											2
*Polyrhachis malaensis*							x																															1
*Polyrhachis nofra*						x																																1
*Polyrhachis osae*				x		x																											x				x	3
*Polyrhachis pacifica*				x																																		1
*Polyrhachis rere*	x			x																						x												3
*Polyrhachis salomo*				x			x															x															x	3
*Polyrhachis santschii*													x	x																								2
*Polyrhachis setosa*				x																																		1
*Polyrhachis similis*						x					x	x	x	x								x					x					x	x					9
*Polyrhachis ugiensis*						x										x																	x					3
*Polyrhachis ulysses*													x	x																		x					x	3
*Polyrhachis undulata*				x		x																																2
*Polyrhachis wheeleri*							x																				x											2
*Polyrhachis* sp. BP01						x																																1
*Polyrhachis* sp. BP03																					x																	1
*Polyrhachis* sp. BP04						x																																1
**Ponera**																																						
*Ponera clavicornis*			x	x		x	x					x	x	x					x	x	x						x	x										12
*Ponera incerta*				x		x						x	x												x	x	x										x	7
*Ponera swezeyi*						x																																1
*Ponera szaboi*		x																			x																	2
*Ponera tenuis*				x																																		1
*Ponera* sp. BP01						x																																1
*Ponera* sp. BP02						x																																1
**Prionopelta**																																						
*Prionopelta majuscula*		x		x	x																																	3
*Prionopelta opaca*		x		x	x	x					x	x	x								x							x	x							x		11
**Pristomyrmex**																																						
*Pristomyrmex levigatus*						x																			x	x												3
*Pristomyrmex obesus*				x		x	x	x			x	x				x									x	x	x											10
**Probolomyrmex**																																						
*Probolomyrmex salomonis*				x		x																															x	2
**Proceratium**																																						
*Proceratium austronesicum*						x																																1
*Proceratium papuanum*																			x			x			x	x												4
**Rhytidoponera**																																						
*Rhytidoponera araneoides*				x		x	x					x	x	x						x							x					x	x				x	10
*Rhytidoponera chalybaea*				x																																		1
**Rogeria**																																						
*Rogeria megastigmatica*				x		x																																2
*Rogeria stigmatica*		x																			x						x						x					4
*Rogeria* sp. BP01*						x																																1
***Romblonella***																																						
*Romblonella elysii*								x					x	x		x		x																				5
**Solenopsis**																																						
*Solenopsis geminata*				x																																		1
*Solenopsis papuana*						x																																1
*Solenopsis pawaensis*						x																					x						x					3
**Stereomyrmex**																																						
*Stereomyrmex dispar*		x																																				1
**Stigmatomma**																																						
*Stigmatomma celata*				x		x	x				x	x	x	x							x						x					x					x	10
*Stigmatomma gnoma*				x																																		1
**Strumigenys**																																						
*Strumigenys chyzeri*				x	x	x	x				x	x															x						x			x		9
*Strumigenys decollata*																											x											1
*Strumigenys emmae*		x		x							x	x								x	x																	6
*Strumigenys eurycera*				x							x	x	x							x																		5
*Strumigenys frivaldszkyi*						x															x																	2
*Strumigenys godeffroyi*				x		x	x	x								x					x	x			x	x	x						x				x	11
*Strumigenys karawajewi*		x		x		x													x							x											x	5
*Strumigenys membranifera*				x		x																			x	x												4
*Strumigenys mocsaryi*				x								x	x																							x		4
*Strumigenys rogeri*				x																																		1
*Strumigenys szalayi*				x		x					x	x								x																		5
*Strumigenys undras*				x																																		1
*Strumigenys yaleopleura*			x								x	x															x							x				5
*Strumigenys* sp. BP05						x																																1
*Strumigenys* sp. BP06						x																																1
**Tapinoma**																																						
*Tapinoma melanocephalum*	x		x	x		x	x						x								x	x				x												9
*Tapinoma minutum*																																					x	0
*Tapinoma timidum*				x																																		1
**Technomyrmex**																																						
*Technomyrmex albipes*			x	x	x	x	x				x	x	x							x	x						x						x				x	12
*Technomyrmex vitiensis*				x		x																																2
*Technomyrmex* sp. BP01						x																																1
**Tetramorium**																																						
*Tetramorium antennatum*																									x	x												2
*Tetramorium aspersum*						x	x				x	x										x	x				x											7
*Tetramorium bicarinatum*	x			x			x									x					x				x	x												7
*Tetramorium carinatum*						x	x	x					x	x		x						x					x					x						9
*Tetramorium insolens*		x				x	x	x								x																	x					6
*Tetramorium lanuginosum*																					x												x					2
*Tetramorium mayri*						x																																1
*Tetramorium melanogyna*						x		x								x																	x					4
*Tetramorium mutatum*							x																															1
*Tetramorium pacificum*		x																			x																	2
*Tetramorium salomo*				x		x	x																															3
*Tetramorium simillimum*				x		x											x				x																	4
*Tetramorium tonganum*				x		x						x								x	x																	5
*Tetramorium vombis*																																	x					1
**Tetraponera**																																						
*Tetraponera laeviceps*				x									x														x											3
**Turneria**																																						
*Turneria dahlii*		x		x			x														x																	4
*Turneria pacifica*						x																			x	x												3
**Vollenhovia**																																						
*Vollenhovia dentata*						x																											x				x	2
*Vollenhovia marginata*							x																										x				x	2
*Vollenhovia elysii*								x								x																					x	2
*Vollenhovia foveaceps*																											x										x	1
*Vollenhovia loboii*							x																														x	1
*Vollenhovia oblonga*						x	x														x				x	x	x											6
*Vollenhovia pedestris*				x		x	x											x							x	x	x											7
*Vollenhovia subtilis*						x	x																															2
*Vollenhovia* sp. BP01*						x																																1
*Vollenhovia* sp. BP02*						x																																1
*Vollenhovia* sp. BP03*																					x																	1
**Wasmannia**																																						
*Wasmannia auropunctata*						x																																1
Total	8	32	11	107	17	142	71	29	7	2	18	40	43	28	4	35	4	8	10	16	66	18	2	4	32	37	68	12	2	1	1	21	38	3	2	7	60	217

## Appendix 1

List of valid species recorded from the Solomon Islands arranged by subfamily, genus and species. (*) Species known to be introduced to the Solomons from outside the Pacific region. ‘Year’ refers to the first year the species was reported from the Solomon Islands. References are arranged in chronological order. Footnotes appended to reference codes indicate that the author misidentified the species or associated it with a different valid name. Reference codes:(1) Forel 1910; (2) Wheeler 1918; (3) Mann 1919; (4) Wheeler 1933; (5) Wheeler 1934; (8) Wheeler 1935a; (9) Chapman and Capco 1951; (10) Wilson 1957; (11) Brown 1958a; (12) Brown Jr. 1958b; (13) Wilson 1958a; (14) Wilson 1958b; (15) Brown 1959a; (16) Brown 1959b; (17) Wilson 1959a; (18) Wilson 1962; (19) Wilson 1959b; (20) Wilson 1959c; (21) Brown Jr. 1960; (22) Brown and Kempf 1960; (23) Taylor 1965; (24) Ettershank 1966; (25) Taylor 1967; (26) Wilson and Taylor 1967; (28) Taylor 1968; (29) Greenslade and Greenslade 1970; (30) Greenslade 1971b; (31) Bolton 1975; (32) Brown Jr. 1975; (33) Brown Jr. 1976; (34) Taylor 1976; (35) Bolton 1977; (36) Greenslade and Greenslade 1977; (37) Brown Jr. 1978; (38) Taylor 1979; (39) Willey and Brown 1983; (40) Ikin 1984; (41) Bolton 1987; (42) Shattuck 1990; (43) Taylor 1991a; (44) Taylor 1991b; (45) Kugler 1994; (46) Brown Jr. 1995; (47) Lin and Wu 1996; (48) Bolton 2000; (49) Ward 2001; (50) Baroni Urbani and De Andrade 2003; (51) Seifert 2003; (52) Wang 2003; (53) LaPolla 2004; (54) Lattke 2004; (55) Kohout 2006; (56) Bolton 2007; (58) Lucky and Sarnat 2008; (59) Seifert 2008; (60) Shattuck 2008; (61) Hosoishi and Ogata 2008; (62) LaPolla 2009; (63) Sorger and Zettel 2009; (64) Kohout 2012; (65) Shattuck and Slipinska 2012; (66) Shattuck et al. 2012; (67) Brown Jr. 1975; (68) Greenslade 1972; (69) Bolton 1985; (70) Forel 1912; (71) Viehmeyer 1924; (72) Donisthorpe 1941; (73) Wetterer 2009; (74) Kohout 1990; (75) Wetterer 1997; (76) Fasi 2009; (77) Foucaud et al. 2010; (78) Fisher and Smith 2008; (79) Collections of Economo and Sarnat 2008; (80) British Natural History Museum, London (Antweb.org records); (81) Australian National Insect Collection, Canberra; (82) Taylor 1980.

## Appendix 2

Presumed undescribed species recorded from the Solomon Islands arranged by species name. The ‘Year’ column refers to the year the species was first recorded from the Solomon Islands. Reference codes are the same as those used in Appendix 11.

## Appendix 3

Occurrence records of individual islands and island groups from which ant species have been recorded arranged by species name and island/island group name. The valid names refer to those presented in Appendix 1. Infraspecific names are abbreviated from trinomials to binomials composed of the genus and infraspecific name (e.g. *Nylanderia obscura bismarckensis* (Forel) is presented as “*Nylanderia bismarckensis”*). Asterisks (*) are appended to morphospecies presumed to be undescribed species (Appendix 2). Morphospecies that we were unable to determine but might represent previously described species are also presented. Individual island names appear in regular type and island group names appear in uppercase bold type. Island groups and their constituent islands from which ants have been recorded are presented in Table 1. The penultimate column ‘Solomon Is.’ includes species records for which no individual island or island group was associated (Brown 1960; 1975; 1976; 1995; Chapman and Capco 1951; Ettershank 1966; Forel 1893; Lin and Wu 1996; Shattuck et al. 2012; Wilson 1959a). The ‘Total’ column sums the number of islands from which each species is recorded, but does not include records from the aforementioned ‘Solomon Is.’ column.
